# The New Pharmacopœia: Its Omissions, Additions, Changes, and Other Features

**Published:** 1914-12-26

**Authors:** 


### THE NEW PHARMACOPŒIA.

### Its Omissions, Additions, Changes, and Other Features.

On January 1, 1915, the standards and formulæ with which prescribers and dispensers have been familiar for the last sixteen years will be superseded by those of the British Pharmacopœia, 1914, and it is, therefore, important that medical practitioners should lose no time in making themselves familiar with the new standards and other special points of the new volume.

### Some Notable Omissions.

The number of omissions from the old Pharmacopœia outweighs the additions to the new, the drugs and preparations omitted being nearly four times the number of those introduced. Perhaps the best known among the discarded drugs are sarsaparilla, gamboge, hops, cantharides, mustard, coca leaves, dandelion, saffron, elder flower, scammony, and musk. No doubt mustard and hops are discarded because the former is a commercial article and the latter is seldom, if ever, used. Sarsaparilla is considered by many to be inert, but, on the other hand, some practitioners still regard it as of value. Cantharides has been replaced by its active constituent cantharidin; coca leaves are also represented by their active principle—namely, cocaine. Scammony is so liable to adulteration that it is preferable to prescribe its active constituent, which has been retained. Gamboge has long been considered too drastic in its effects.

### Some of the Additions.

Notwithstanding the vast array of new drugs—mostly of German origin—that have been introduced during the past sixteen years, the committee of revision has considered it necessary to introduce only forty-three drugs and preparations that were not previously recognised officially. The most important of the additions are adrenalin, acetylsalicylic acid (which was first introduced under the trade-mark name Aspirin), hexamethylenetetramine (for which the short name Hexamine has been made official), diethyl-barbituric acid (which was first introduced as veronal but for which the new name is Barbitone), diamorphine hydrochloride, benzamine lactate ($\beta$-eucaine lactate), chloral formamide (chloralamide), methyl sulphonal, methyl salicylate, phenolphthalein, resorcin, and senna pods.

### Changes in Potency.

The strength of forty-one preparations has been changed, the reason being in some cases (*e.g.* the tinctures of belladonna, digitalis, aconite, nux vomica, and opium) to promote uniformity in the Pharmacopœial usage of different countries. The most notable alteration in the strength of a potent preparation is in the case of tincture of strophanthus; in the new Pharmacopœia the strength of this tincture is practically the same as that recommended by the International Congress on the uniformity of potent drugs, and is four times that of the tincture of the Pharmacopœia of 1898. The dose has been reduced to one-third, and practitioners will have to exercise care in prescribing it; it might be advisable for practitioners to indicate on their prescriptions "B.P., 1914," so that the dispenser will have no doubt that the new tincture is meant; even if the new Pharmacopœia is not indicated pharmacists will be expected to dispense the new tincture, but it might be as well to adopt this measure of precaution. Tincture of aconite is approximately twice as strong as the old tincture. Tincture of opium has been increased in strength by about one-third, and paregoric by about 10 per cent. Syrup of chloral has been increased in strength by about 10 per cent., and syrup of codeine to the same extent. Vinegar of squills is approximately twice as strong as formerly. Calomel ointment is double the old strength. Tincture of colchicum, carbolic acid lozenges, tincture of nux vomica, belladonna plaster, hypodermic injection of cocaine, and hypodermic injection of morphine have all been reduced to one-half strength. Liniment of mercury contains three-fifths of the proportion of mercury. Syrup of iodide of iron is about 30 per cent. weaker, and tincture of belladonna has been reduced in strength to about the same extent. Tincture of digitalis is 20 per cent. weaker. Carbolic acid ointment is 25 per cent. weaker. Solution of mercuric chloride has been reduced in strength from 1 in 875 to 1 in 1,000. Tablets of trinitro-glycerin contain 0.5 milligram in each instead of 0.65. Ammoniated mercury ointment has been reduced in strength to one-half. Phosphorus pill contains 1 per cent. of phosphorus instead of 2. Spirit of nitrous ether may now contain a minimum percentage of 1.52 of ethyl nitrite instead of 2. Mercury ointment has been reduced to three-fifths its old strength, and compound mercury ointment to the same extent.

### Other Features of the New Work.

With regard to weights and measures the metric system alone is employed for all pharmaceutical and analytical computations, but doses are given in both the metric and imperial weights and measures. A note in the preface states that the doses given in the Pharmacopœia are not authoritatively enjoined by the General Medical Council as binding upon prescribers, and it is recommended that practitioners should cease to use the usual symbols for the ounce and the drachm; the reason for this recommendation is that the symbol ʒ is often used to represent 60 grains and also to represent the fluid drachm, and the symbol ℥ to represent 480 grains, sometimes 437.5 grains, and also to represent the fluid ounce. Limits have been fixed to the proportion of lead and arsenic permissibly present as impurities in about ninety substances. The number of crude drugs and their preparations which are required to contain a definite proportion of the chief active constituents has been materially increased.

